# Editorial: Tumor and immune cell interactions in the formation of organ-specific metastasis

**DOI:** 10.3389/fonc.2024.1373308

**Published:** 2024-02-20

**Authors:** Tabea Gewalt, Linda Diehl, Lydia Meder

**Affiliations:** ^1^ Department I of Internal Medicine, Faculty of Medicine and University Hospital Cologne, University of Cologne, Cologne, Germany; ^2^ Institute for Experimental Immunology and Hepatology, University Medical Center Hamburg-Eppendorf, Hamburg, Germany; ^3^ Hamburg Center of Translational Immunology (HCTI), University Medical Center Hamburg-Eppendorf, Hamburg, Germany; ^4^ Department of Experimental Medicine 1, Medical Faculty, Friedrich-Alexander University Erlangen-Nürnberg, Erlangen, Germany

**Keywords:** metastasis, microenvironment, solid cancer, immune cells, cellular crosstalk

Metastasis represents the most disastrous stage of malignant cancer diseases and is associated with a worse clinical prognosis of cancer patients. The metastatic process includes different subsequent steps including dissemination of tumor cells from the primary site, intravasation into the blood stream and survival in the circulation, extravasation and finally colonization of the metastatic site. Primary cancer cells frequently follow a preferred metastasis pattern and invade distinct distant organs, also known as “organ-specific metastasis” or “organotropism”. One factor determining organotropism is the interaction between cancer cells and the host immune cells (1, 2). These interactions can appear as an indirect cellular communication via soluble factors or as direct cell-cell contacts. Both kinds of cellular crosstalk are known to program the tumor microenvironment (TME) enhancing metastases formation (3, 4).

In the Research Topic *Tumor and immune cell interactions in the formation of organ-specific metastasis*, five articles were recently published highlighting five types of cell-cell interactions in the TME in organotropic metastasis formation.

The review of Xie et al. described the involvement of the different immune cell types in the microenvironment of brain metastasis in non-small cell lung cancer (NSCLC). Particularly, they discussed the clinical advancement of immune checkpoint inhibitor (ICI) treatment concerning brain metastasis.

Microglia and bone marrow-derived macrophages (BMDMs) were mentioned as major players in brain metastasis. Depending on their immune status, they secreted cytokines related to the M1 or the M2 phenotype. The tumor-suppressing M1 phenotype secreted pro-inflammatory cytokines, such as interleukin-6, tumor necrosis factor α or reactive oxygen species to fight the cancer cells and thus to prevent metastatic growth. Besides, the pro-inflammatory cytokines also supported their phagocytic activity and the integrity of the blood-brain-barrier. However, the switch to the M2 phenotype including the secretion of anti-inflammatory cytokines, like interleukin-4 and -10, had the opposite effect and tended to promote metastatic development. The exact mechanisms of M2 phenotype induction needs to be unraveled, as a switch back to the M1 phenotype may improve the therapeutic outcome for NSCLC patients with brain metastasis. A first step may be the deciphering the spatial transcriptomic landscape of brain metastasis in NSCLC, as recently described (5).

Pontis et al. focused in their review on the role of extracellular vesicles (EVs) in the pre-metastatic niche (PMN) formation in lung cancer metastasis. By carrying certain miRNAs to target sites, EVs from lung and breast primary tumors interacted with endothelial cells and foster vascular permeability. Moreover, EVs were able to activate fibroblasts, supporting colonization of cancer cells. In addition, EVs can have immunomodulatory effects by delivering immune related cargo to the PMN. It has been shown that exosomes secreted from LIN28B-high primary breast cancer cells were able to establish an immune-suppressive lung PMN and thus encouraged organ-specific metastasis (6). Of note, LIN28B is an RNA-binding protein enhancing tumor aggressiveness and metastatic properties in several solid cancers by regulating miRNA-dependent pathways (7). Pontis et al. pointed out the interaction between EV’s PD-L1 and the PD-1 on cytotoxic T-cells contributing to the inactivation of T-cells so that a supportive TME for tumor and metastasis growth was created.

Taken together, EVs may indirectly influence the aggressiveness and the dormancy state of cancer cells promoting their extravasation by modulating immune and stromal cells.

As a third pillar, Li et al. characterized in their review the properties of the TME leading to lymphatic metastasis in cervical cancer. They highlighted five factors, which facilitated lymphatic metastasis: Immunosuppression, PMN formation, lymphangiogenesis, epithelial-to-mesenchymal transition (EMT) and extracellular matrix (ECM) remodeling. Of note, compounds targeting angiopoietins to affect lymphangiogenesis (8) in cancer have been tested in combination with ICI for example in NSCLC (9). Finally, Li et al. aim for deciphering the responsible mechanisms in cervical cancer lymphatic metastasis in order to implement this finding into clinical applications.

A fourth important constituent in promoting metastasis is the incidence of “fusion hybrids”. The review of Cozzo et al. discussed this phenomenon in the context of organ-specific metastasis. The homotypic fusion described the fusion between cancer cells, whereas heterotypic fusion referred to the fusion of cancer cell and immune cell with monocytes and macrophages as preferred fusion partner. Hybrid fusion entailed chromosomal instability and genome rearrangement leading to a greater tumor heterogeneity. Through adapting characteristics from both parental cells, the hybrid fusion cells tended to be more invasive and aggressive and were equipped with a higher capability to escape immunosurveillance. This has been highlighted in hybrids of primary lung cancer cells and monocytes. Here, hybrids were capable of suppressing T cells in a PD-L1-dependent manner and attenuating the perforin secretion level of NK cells (10).

These gained capabilities of hybrid cells in the TME offer them a selection advantage and increased metastatic potential.

Huang et al. performed an integrated single-cell transcriptomic analysis of the myeloid compartment in lung-specific metastasis of breast cancer. The results disclosed four distinct myeloid subpopulations comprising monocytes, macrophages, dendritic cells and neutrophils in the lung microenvironment having promoting effects on organ-metastatic progression. The aspect of macrophage plasticity in organ colonization and metastatic outgrowth has been also described in other organs such as the liver (11).

Huang et al. identified the following myeloid cell subsets contributing to lung metastasis: *Tppp3^+^* monocytes supported lung metastasis by enhancing angiogenesis, *Isg15^+^* macrophages and *Il12b^+^* dendritic cells had their promoting effect via fostering immunosuppression in the lung, and metastasis promoting *Ifit3^+^* neutrophils with a unique expression profile.

Finally, a cell-cell communication analysis showed that these populations also interacted with each other to influence their polarization and pro-tumorigenic activity. This study clearly demonstrated that the myeloid heterogeneity and interaction created a tumor-supporting environment pivotal in the progression of organ-specific metastasis.

The collected articles discussed cellular communication via cytokines, EV secretion, lymphatic vessel metastasis, the incidence of hybrid fusion cells and the myeloid heterogeneity at the metastatic site as aspects of organotropism (**Figure 1**). It is required to deepen our understanding of the molecular interactions between host cells and tumor cells at their primary site of origin and at metastatic sites. Unraveling the disparities in the cellular crosstalk at different sites of colonization can contribute to novel therapeutic strategies for metastatic cancer in the future.

**Figure 1:**
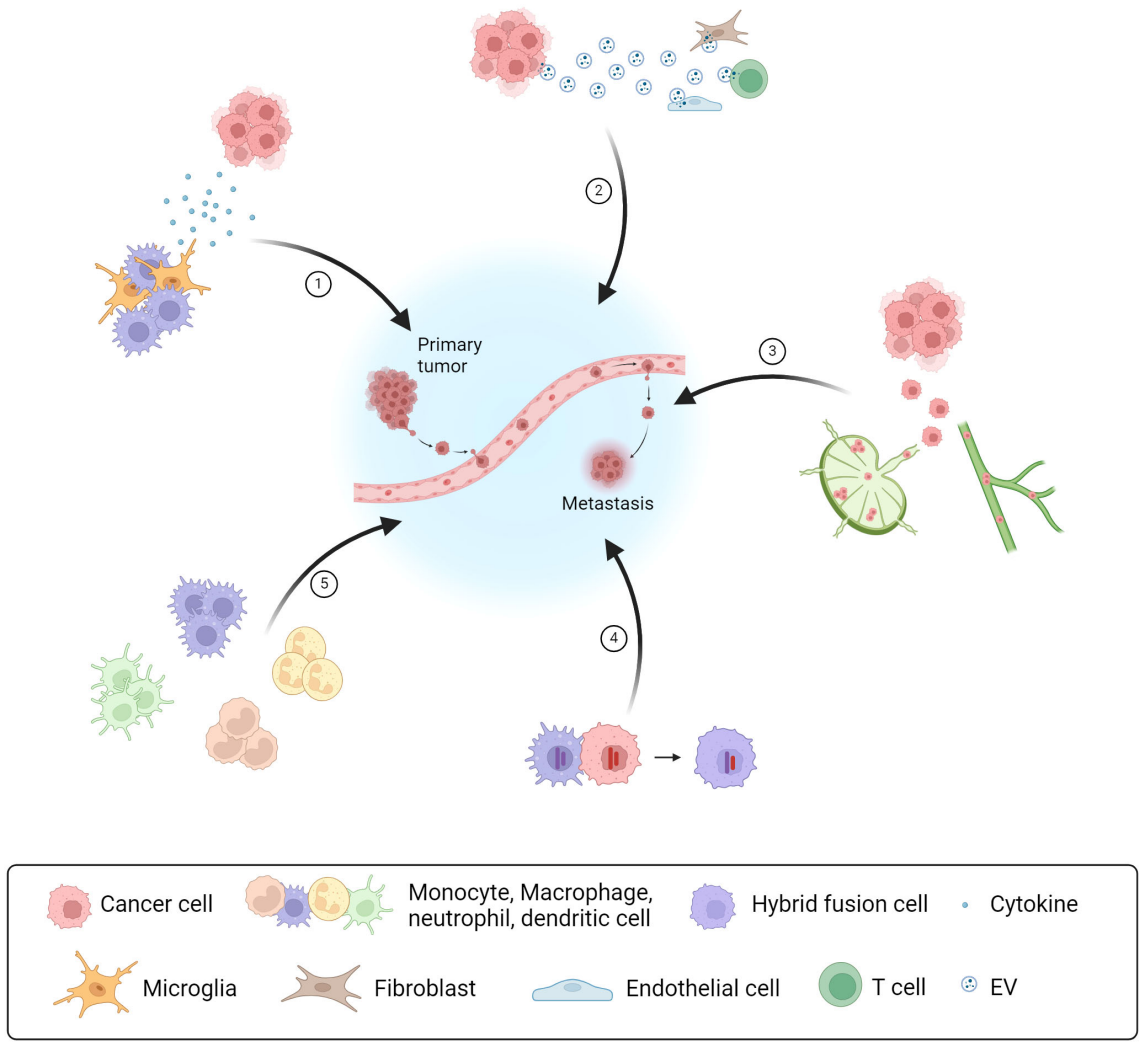
Types of intercellular crosstalk in the formation of organ-specific metastasis Overview of five cellular communication strategies as part of the intercellular crosstalk in the TME between cancer and host immune cells or stromal cells, which promote PMN formation and metastasis: (1) Communication via soluble factors, like cytokines secreted by host immune cells; (2) Communication via EVs released by cancer cells modulating the activity of different immune cells and stromal cells; (3) Interaction of cancer cells via the lymphatic system and lymphatic vessel metastasis; (4) Direct cell-cell contact and fusion with host immune cells resulting in hybrid fusion cells; (5) Myeloid heterogeneity in the TME having different promoting effects for metastasis and cancer progression. Created with BioRender.com.

## Author contributions

TG: Writing – original draft, Writing – review & editing, Visualization. LD: Writing – review & editing. LM: Supervision, Writing – original draft, Writing – review & editing.
